# Ultrasound Sonosensitizers for Tumor Sonodynamic Therapy and Imaging: A New Direction with Clinical Translation

**DOI:** 10.3390/molecules28186484

**Published:** 2023-09-07

**Authors:** Yunlong Liang, Mingzhen Zhang, Yujie Zhang, Mingxin Zhang

**Affiliations:** 1 Second Clinical Medical College, Shaanxi University of Chinese Medicine, Xianyang 712046, China; ylongliang0608@163.com; 2 School of Basic Medical Sciences, Xi’an Jiaotong University Health Science Center, Xi’an 710061, China; mzhang21@xjtu.edu.cn; 3 Department of Gastroenterology, The First Affiliated Hospital of Xi’an Medical University, Xi’an 710077, China

**Keywords:** sonodynamic therapy, sonosensitizers, clinical, integrated diagnosis and treatment

## Abstract

With the rapid development of sonodynamic therapy (SDT), sonosensitizers have evolved from traditional treatments to comprehensive diagnostics and therapies. Sonosensitizers play a crucial role in the integration of ultrasound imaging (USI), X-ray computed tomography (CT), and magnetic resonance imaging (MRI) diagnostics while also playing a therapeutic role. This review was based on recent articles on multifunctional sonosensitizers that were used in SDT for the treatment of cancer and have the potential for clinical USI, CT, and MRI applications. Next, some of the shortcomings of the clinical examination and the results of sonosensitizers in animal imaging were described. Finally, this paper attempted to inform the future development of sonosensitizers in the field of integrative diagnostics and therapeutics and to point out current problems and prospects for their application.

## 1. Introduction

Despite enormous efforts to cure cancer, it is still the most serious health problem human beings face in the 21st century due to its high morbidity and mortality [1]. Presently, there are four primary approaches that have received approval for the clinical management of cancer: surgery, chemotherapy, radiotherapy, and immunotherapy [2]. However, these treatments are also associated with high recurrence rates and uncontrollable adverse effects. Therefore, what is the future of anti-cancer therapy with high efficiency and low medical costs? Recently, several non-invasive cancer treatment modalities have attracted the attention of researchers worldwide, among which photothermal therapy (PTT), photodynamic therapy (PDT), and sonodynamic therapy (SDT) are representative methods [3,4,5]. PTT induces necrosis or decomposition of cancerous tissue by activating the energy converted through the photothermal agents (PTAs) [3,4,5]. PTT converts the absorbed light energy into heat energy by activating photothermal agents (PTAs) to induce necrosis or disintegration of cancer tissue. Although PTT has been recognized for its efficacy in the treatment of cancer since the 19th century, it is poorly targeted to cancer patients due to photothermal agents (PTAs). PTT may cause unnecessary medical harm by causing damage to healthy tissues unrelated to treatment [6,7]. Both PDT and SDT are considered high-precision and non-invasive treatment modalities. PDT is a treatment based on the conversion of light energy absorbed by photosensitizer molecules into reactive oxygen species (ROS), thereby mediating tumor cell death. However, since light can only penetrate a few millimeters of tumor tissue, PDT is only suitable for some superficial tumors.

As the research progresses, the benefits of SDT in the treatment of tumors have attracted our strong interest. SDT is a non-invasive treatment that does not require incisions or punctures, reducing surgical risks and recovery time. SDT can maximize the protection of surrounding healthy tissue by adjusting the energy and depth of focus of ultrasound. Compared with radiation therapy or chemotherapy, SDT treatment does not produce radiation, reducing the damage to surrounding normal tissues. Depending on the patient, SDT allows for individualized treatment plans to be designed to improve outcomes. Because SDT has so many advantages, its use in oncology has been widely studied. The research and development were simultaneously able to treat and provide imaging guidance, providing valuable direction for multimodal therapeutic applications of cancer treatment [8].

This paper began with a comprehensive summary of the mechanics of SDT. Next, it will focus on sonosensitizers with clinical translation potential and integrated diagnostic and therapeutic capabilities. Finally, the clinical application of diagnostic and therapeutic integrated sonosensitizers, as well as the future development direction, were prospected.

## 2. Overview of SDT

The origins of SDT can be traced back to the evolution of PDT. PDT is a technology that uses the photodynamic effect produced by photosensitizers for disease diagnosis and treatment. The process is that light at a specific wavelength (600–800 nm) activates a photosensitizer in the target tissue, which in turn transmits the received energy to oxygen molecules in an excited state in the tissue, thereby inducing the generation of ROS. Singlet oxygen (^1^O_2_), hydrogen peroxide (H_2_O_2_), and hydroxyl radicals (·OH) are the main components of ROS. Reactive oxygen species produce cytotoxic effects, leading to cell damage and even death [9,10]. SDT shares similarities in mechanism with PDT, but it uses acoustic energy rather than light energy. SDT has several advantages over PDT. The penetration depth of ultrasound into biological tissues is much deeper than that of light [11]. Therefore, SDT can be used to treat deep tumors, such as those in the liver, brain, and pancreas [12,13]. In addition, the cavitation effect of ultrasound can cause mechanical damage to cells, and ultrasound can also change the fluidity of the phospholipid bilayer, thereby changing the membrane permeability [14]. Besides, the acoustic force has lower operating and instrument costs. Furthermore, since visible light can activate most of the photosensitizing chemicals used in PDT therapy, patients may experience photosensitizing allergic reactions after the use of photosensitizers that may affect the quality of their survival [15]. It is generally accepted that SDT is a method of producing cytotoxic effects by means of acoustic sensitizers with very low biotoxicity [16]. Therefore, SDT is a more promising tumor treatment strategy than PDT.

SDT, as a non-intrusive means, can kill cancer cells precisely by controlling the intensity, frequency, and time of ultrasound irradiation to activate the sonosensitizers accumulated at the tumor site. The cytotoxic effects of SDT were first reported in 1989, when researchers observed that the combination of US and the photosensitizer hematoporphyrin produced cytotoxic effects on mouse sarcoma cells [17]. Subsequently, the phenomenon of hematoporphyrin producing cytotoxic effects under ultrasonic irradiation was named the “sonodynamic method” [18]. In recent years, various types of sonosensitizers, including porphyrins, phthalocyanines, chlorines, and a variety of acoustic nanomaterials, have been discovered that can generate ROS and exert cytotoxic effects triggered by US irradiation [19,20]. The toxic effects of SDT on tumor cells can be achieved by activating apoptosis and immunity and affecting the tumor microenvironment. Studies have shown that the generated ROS in SDT treatment caused a decrease in mitochondrial membrane potential, which led to damage to mitochondria, and then cytochrome c (Cyt c) was released from the mitochondria into the cytoplasm, and finally the caspase-dependent apoptotic pathway was activated [21,22]. Honda et al. [23] reported that disrupted mitochondrial membrane potential released ROS, which further activated Caspase-3 and ultimately led to the induction of DNA breaks. The growth and metastasis of cancer cells depend on the tumor microenvironment. Over the past few years, several studies have revealed that the tumor microenvironment is regulated by multiple factors, including SDT treatment. Gao et al. [24] found that SDT can induce ROS production in the tumor microenvironment, which can significantly inhibit the proliferation, migration, and invasion of endothelial cells. In addition, SDT can activate immune responses via SDT-injured tumor debris or immune response complexes in the tumor microenvironment. The results showed that M2 macrophages in the tumor were converted to the M1 phenotype during SDT treatment. Meanwhile, dendritic cells (DCs) in the tumor microenvironment tended to mature as the expression levels of CD_80_ and CD_86_ molecules increased in activated T cells [25]. SDT treatment promotes the proliferation of cytotoxic T-lymphocytes (CD8^+^ T-cells) in tumors by upregulating the expression of CRT, HMGB1, and HSP70 [26].

## 3. Mechanisms of SDT

**Figure 1:**
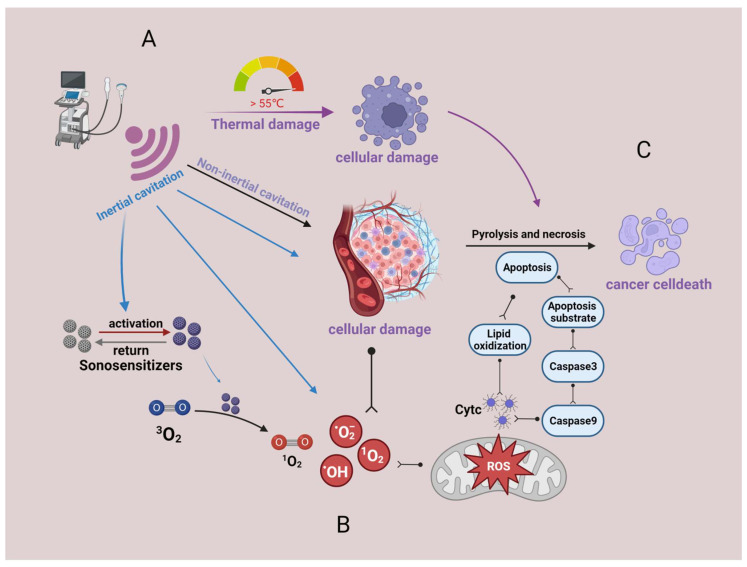
Principles of SDT for oncology treatment. (**A**) Ultrasound carries acoustic energy that can produce both thermal and non-thermal effects in the tumor region. The thermal effect can directly cause cellular damage. Non-thermal effects mainly occur as cavitation effects. (**B**) ROS generation. Ultrasound induces intracellular ROS generation inside the tumor by producing cavitation effects and activating sonosensitizers. (**C**) ROS cause tumor cell death by inducing cellular autophagy and necrosis, among other pathways. Created with BioRender.com (accessed on 23 August 2023).

Thermal and non-thermal effects may be the main pathways through which SDT works [16]. Figure 1 depicts the principle of SDT operation.

### 3.1. Thermal Damage

When tissues are irradiated by ultrasound, some of the energy carried by the sound waves is converted into heat energy. If the temperature of the tissue exceeds the threshold (56 °C), it becomes thermotoxic, leading to irreversible coagulative necrosis of the cells. It has been developed as a strategy to focus high-energy ultrasound on the lesion for the thermal ablation of tumors [27]. High-intensity focused ultrasound (HIFU) has been used in clinical disease since the latter half of the 1990s and achieves thermal coagulation in less than 12 s. In clinical practice, HIFU treatment uses conventional frequencies ranging from 0.8 to 3.5 MHz. High-frequency ultrasound between these frequencies carries a higher energy beam than diagnostic ultrasound. At lower deposition energy doses (<55 °C), the induced hyperthermia increases the cell permeability to facilitate nanoparticle delivery effectively [28]. This is advantageous when used in conjunction with thermally modulated carrier molecules. At higher doses of deposition energy (>55 °C), cells die due to coagulative necrosis [29].

### 3.2. Non-Thermal Effects

The non-thermal effects of ultrasound can be categorized as follows: (i) ultrasonic cavitation effect; (ii) generation of ROS; and (iii) ultrasound-induced apoptosis of cancer cells.

#### 3.2.1. Ultrasonic Cavitation Effect

When intense ultrasound waves irradiate tissue fluid, much of the microbubbles are produced and collapse, a process known as cavitation, which contains inertial cavitation and non-inertial cavitation in general [30,31]. The inertial cavitation process includes the stages of nucleation, expansion, oscillation, and implosive collapse of microbubbles in liquids, which process generates localized extremely high temperatures (>10,000 K) and pressures (>81 MPa) as well as free radicals [32]. Non-inertial cavitation, sometimes called “stable cavitation,” is a sustained linear or nonlinear oscillation around the equilibrium radius of an acoustically driven bubble [33]. Stable cavitation leads to microflows characterized by the generation of fluid flow [28]. However, microfluidics can lead to cell lysis and death [34]. Also, the stable cavitation generates heat due to the loss of viscosity at the bubble surface boundary. As these extreme physical conditions dramatically alter the environmental balance within the tissue, irreversible damage to cellular structures is induced, ultimately leading to cellular necrosis.

#### 3.2.2. Generation of Reactive Oxygen Species

The production of ROS, including singlet oxygen (^1^O_2_), hydroxyl radicals (OH), peroxides, and superoxide (O_2_^−^), is the main source of cytotoxicity in SDT. It has been suggested that the interaction of ultrasound with different types of sonosensitizers to produce ROS may involve acoustic cavitation, acoustoluminescence, and pyrolysis [2]. Although different types of cavitation effects produce mechanical effects, acoustic-chemical species are only produced by inertial cavitation [35]. Inertial cavitation is an ultra-violent process of nanometer bubble activity at a microsecond level, usually accompanied by extremely high temperature (>10,000 K) and pressure, which leads to the pyrolysis of water vapor within the microbubble, generating highly reactive hydroxyl radicals and hydrogen atoms [35]. It was reported that when receiving ultrasonic energy, nanobubbles can behave as cores for inertial cavitation, thereby inducing ROS production [36]. After receiving acoustic energy from ultrasonic cavitation, the sonosensitizers are mobilized from the basal state to the exciting mode, and then the activated sonosensitizers return to the basal state, releasing energy and reacting directly with the surrounding oxygen to produce single-linear oxygen [35]. As the cavitation bubble expands and eventually collapses, a transient hot spot of high temperature and high pressure is formed, generating self-luminescence light from ultraviolet (UV) to visible light, called sonoluminescence. Studies have shown that some sonosensitizers with photosensitizing properties are able to absorb and convert the light generated during ultrasonic cavitation into heat and reactive oxygen species [37]. The sonosensitizers were activated by sonoluminescence and finally transferred electrons to O_2_, generating cytotoxic ROS, which can induce membrane lipid peroxidation, cell membrane instability, and irreversible cellular damage [38,39]. However, for some sonosensitizers that lack photosensitivity, ROS cannot be generated via the acoustoluminescent pathway. Kessel et al. [40] showed that ultrasound-mediated inertial cavitation of microbubbles on the liquid surface at high localized temperatures induced the decomposition of ultrasound sensitizers. The widely accepted theory suggests that ROS generated during inertial cavitation can be directly pyrolyzed or chemically reacted with sonosensitizers to generate free radicals, which further react with other endogenous substances to generate more ROS [41].

## 4. Integration of Diagnosis and Treatment

**Figure 2:**
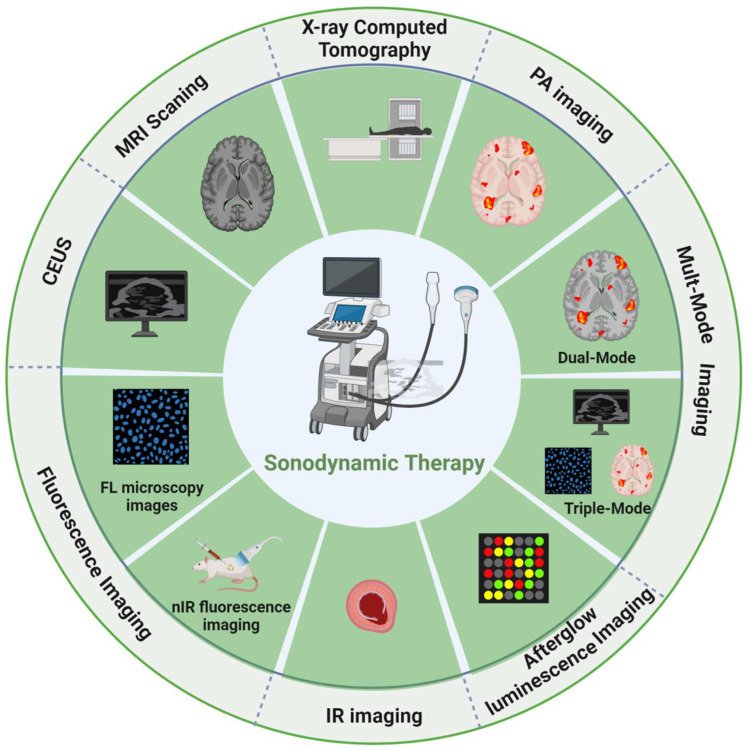
Summary of the integrated diagnosis and treatment scheme involving sonodynamic therapy. MRI: magnetic resonance imaging. CEUS: Contrast-Enhanced Ultrasound. Fluorescence imaging includes FL microscopy images [47] and near infra-red (nIR) fluorescence imaging [50]. IR: infrared image [53]. Afterglow luminescence image [54]. Mult-Mode image contains Dual-mode and Triple-mode. PA: Photoacoustic imaging [55]. Created with BioRender.com (accessed on 23 August 2023).

Therapeutics refers to treatment strategies that combine diagnostic tests with specific treatments based on test results, a term derived from “diagnosis” and “treatment” [42]. This capability to integrate imaging and therapy is critical for the future treatment of cancer. As a result, sonosensitizers have recently received extensive research in the field of cancer diagnosis and treatment [43]. In recent years, with the development of novel nanoparticles (NPs), some multifunctional sonosensitizers can not only treat tumors but also play the function of imaging, including MRI, USI, CT, photoacoustic imaging (PAI), and fluorescence imaging (FLI), realizing the integration of cancer diagnosis and therapy [44,45,46,47,48,49,50] (Figure 2). In cancer diagnostics, many multifunctional sonosensitizers can be used as contrast agents (CA) for imaging examinations, which is easily achieved due to their targeted nature and specific enrichment in tumor tissues [51,52]. Compared with conventional scanning techniques, nanoparticle-based contrast agent micrography features noninvasive, instant monitoring, targeting, and high spatial and temporal resolution, which provides a basis for accurate diagnosis and visual tracking during healing. In tumor therapy, sonosensitizers as imaging contrast agents play a role in treating tumors while visually tracking their accumulation in tumor tissue with the help of their imaging capabilities. Comparison of the gray-scale changes of tumor tissues and their quantitative gray-scale values in the imaging maps of the imaging instruments (CT, US, and MRI) before and after SDT treatment allows a visual and easy assessment of the treatment effect [8,48]. In addition, the use of ultrasound bio-imaging capabilities and continuous tracking of drug retention time in the tumor allows for accurate and timely information during treatment, thus improving the accuracy of SDT and the optimal duration of treatment after i.v. administration. Therefore, combining these sonosensitizers with clinical examination can effectively improve the accuracy of image information and facilitate clinical analysis and treatment. This review focuses on three imaging modalities (MRI, USI, and CT) mediated by sonosensitizers and their basic principles, aiming at a better understanding of sonosensitizers for imaging and therapeutic use and looking ahead to the challenges of clinical translation.

## 5. Sonosensitizers with Various Imaging Functions

### 5.1. Contrast-Enhanced Ultrasound (CEUS)

Ultrasound (US) is a sound wave with a frequency of more than 20,000 Hz that is inaudible to the human ear. Ultrasound has the advantages of low-cost, simple, rapid, non-invasive, non-radioactive, accurate, continuous, dynamic, and repeatable scans [43,56,57]. Using the physical properties of ultrasound, various cross-sectional images of organs and surrounding organs can be displayed, which is close to the anatomical real structure. Therefore, ultrasound is often used as the first choice for the examination of solid organs and fluid-containing organs. In particular, ultrasound elastography and contrast-enhanced ultrasound (CEUS) are well established as being used for diagnosis. In CEUS, intravenously injected microbubbles are excited by longitudinal ultrasound in the examined area, producing nonlinear oscillations. The corresponding contrast agent software can distinguish the diseased tissues from the received contrast agent signals [58]. However, currently, 78.5% of radiology departments use diagnostic ultrasound imaging as a routine diagnostic imaging method, while only 26% of them use contrast-enhanced ultrasound [58]. The excessively expensive price of ultrasound contrast agents and their lower selectivity limited the clinical application of CEUS. It was found that some sonosensitizers used for SDT treatment also showed promising results in CEUS. Table 1 lists the sonosensitizers and imaging capabilities used for CEUS imaging. Sonosensitizers produce stable microbubbles (MBs) or nanobubbles (NBs) under CEUS cavitation to achieve enhanced imaging [59,60,61]. Sonosensitizers achieve synergistic drug delivery and tumor therapy by affecting the lesion’s tissue structure. Therefore, with the development of sonosensitizers, the clinical application of contrast-enhanced ultrasound is becoming more and more broad.

**Figure 3:**
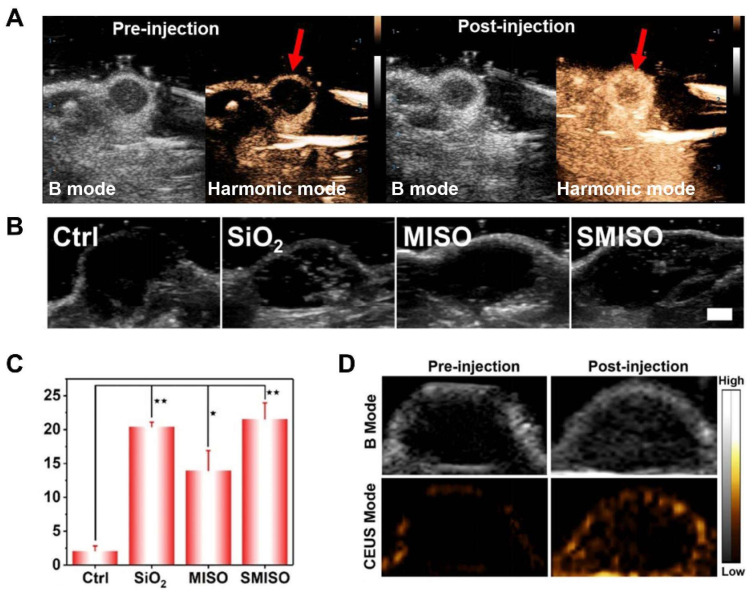
(**A**) Grayscale and color US images (B mode and Harmonic mode) of TRAMP cells implanted in the tumor before (left) and after (right) injection of PCF-MBs. The red arrows highlight the tumor sites. (**B**) In vivo B-mode US imaging. (4T1 tumor-bearing nude mice). (**C**) Corresponding grayscale values of tumors obtained 5 min after intratumoral injection of PBS, SiO_2_ (10 mg/kg), MISO (5 mg/kg), and SMISO (15 mg/kg) (* *p* < 0.05, ** *p* < 0.01, n = 3, mean ± SD). Scale bar: 2 mm. (**D**) Before and after comparison of B-mode and CEUS-mode imaging with ultrasound irradiation. Notes: (**A**) Adapted with permission from Ref. [49]. Copyright 2018, American Chemical Society. (**B**,**C**) Adapted with permission from Ref. [66]. Copyright 2023, American Chemical Society. (**D**) Adapted with permission from Ref. [47]. Copyright 2019, American Chemical Society.

Chen M. et al. [49] reported a liposomal nanoparticle based on porphyrin/camptothecin-floxuridine triad microbubbles (PCF-MBs). The novel PCF-MB not only realized ultrasound imaging but also achieved chemo-photodynamic combination therapy. After intravenously injecting 1 mg/mL of PCF-MBs into Balb/c nude mice bearing HT-29 colon cancer, the ability of PCF-MBs to potentiate ultrasound imaging was investigated by a fixed-frequency ultrasound transducer. The results showed that before the injection, there was hardly any sonographic signal in the tumor. The ultrasound imaging signals peaked 20 s after injection, and the peak state lasted more than 3 min (Figure 3A).

Zhang et al. [66] synthesized a composite system in which Mn-doped In_2_S_3_/InOOH (SMISO) is loaded in spinodal silica (r-SiO_2_) to integrate ultrasound imaging and SDT for the detection and treatment of breast cancer. The live Type B US imaging results showed that the grayscale was changed after injection of SiO_2_, MISO, or SMISO solutions in the 4T1 breast tumor model, and there was a statistical difference between SMISO and the control group (*p* < 0.01), suggesting SMISO could achieve ultrasound imaging (Figure 3B,C). Further, it was found that the signal intensity increased after SMISO NPs injection and peaked at 12 h, guiding the optimal time for ultrasound irradiation. And then, 12 h after the NPs injection, the signal intensity gradually decreased until it disappeared, indicating that the NPs were eliminated from the tumor. Under US irradiation, the SMISO NPs effectively inhibited tumor growth. In summary, the nanoplatform simultaneously had therapeutic efficacy and imaging capability.

Zhang L et al. [47] fabricated an innovative nanoplatform capable of multimodal (FLI/PAI/USI) imaging using IR780 and perfluoropentane (PFP), as well as guiding SDT for tumor treatment. After intravenous injection of IR780-NDs, researchers acquired enhanced ultrasound images of xenograft tumors in nude mice with 4T1 breast cancer. At 24 h after administration in the caudal i.v., the US signal in CEUS mode showed brightness (Figure 3D). In addition, the quantification data indicated that the echo intensity was much stronger in the post-injection group than in the pre-injection group.

Ho et al. [45] designed multi-functional superhydrophobic mesoporous silica nanoparticles (FMSNs) for the encapsulation of the clinical anti-cancer drug Doxorubicin (FMSNs-Dox). In this study, monodisperse silica super-hydrophobicity was utilized to significantly improve the contrast of ultrasound images by enhancing the accumulation of interfacial nanobubbles (INBs). FMSNs-Dox possessed excellent ultrasound imaging ability, an anti-vascular effect, and antitumor treatment capability under ultrasound irradiation. US images showed that the contrast was significantly enhanced by INBs in tumor tissue from 1–9 days.

### 5.2. X-ray Computed Tomography (CT)

CT scanning utilizes a computer to process a combination of several X-ray images acquired from different angulations to develop an anatomical picture of the scanned object [73]. CT has become a popular noninvasive clinical imaging method because of its reproducibility, low price, and ease of use [74]. CT scans are divided into conventional and enhanced scans according to whether contrast media is used or not. Since plain CT cannot distinguish between tissues with similar mass attenuation coefficients (e.g., normal organs and tumors), exogenous X-ray attenuating CT contrast materials need to be injected intravenously to identify diseased tissues. Iodine contrast is commonly used in clinical practice but has the following limitations: (1) susceptibility to allergic reactions and nephrotoxicity; (2) low dose-efficiency ratio; and (3) lack of targeting. Studies have investigated the utilization of sonosensitizers as CT imaging agents to overcome the shortcomings of commonly used clinical contrast agents. Table 2 lists the sonosensitizers and imaging capabilities used for CT imaging.

**Figure 4:**
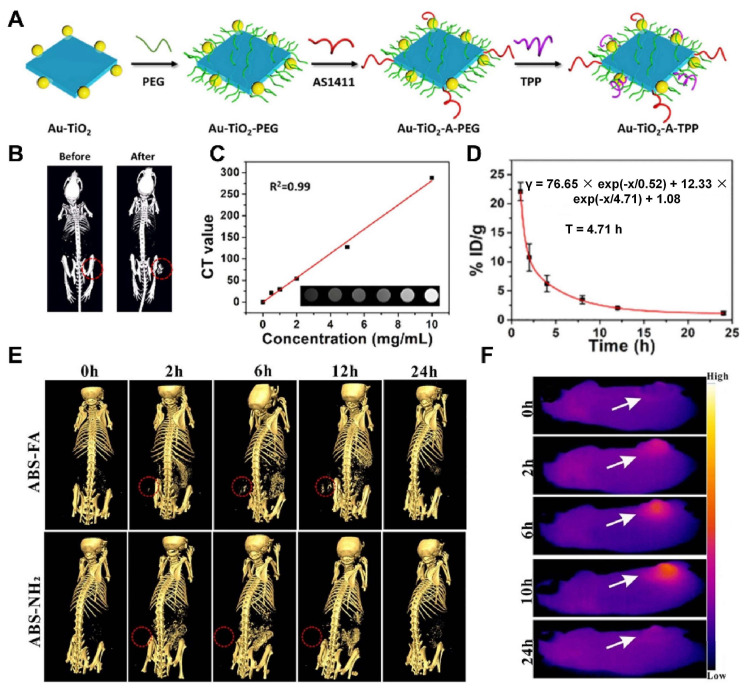
(**A**) The process of synthesis of Au-TiO_2_-A-TPP. (**B**) Comparison of CT images of mice before and after intravenous injection of Au-TiO_2_-A-TPP (10 mg/kg). The red circles highlight the tumor sites. (**C**) In vitro CT imaging and CT signal values about different concentrations of Au-TiO_2_-A-TPP. (**D**) Blood circulation time in MCF-7 tumor-bearing mice after intravenous injection of Au-TiO_2_-A-TPP (10 mg/kg). (**E**) CT imaging of HeLa tumor-bearing mice after in vivo injection of ABS-FA and ABS; red dashed circle: tumor sites. (**F**) Low power (0.35 W/cm^2^) infrared thermograms of tumor-bearing mice taken at different time points. The white arrows highlight the tumor sites. Notes: (**A**–**D**) Adapted with permission from Ref. [51]. Copyright 2019, American Chemical Society. (**E**,**F**) Adapted with permission from Ref. [48]. Copyright 2020, American Chemical Society.

Cao et al. [51] designed and synthesized titanium oxide (TiO_2_) nanosheets with triphenylphosphine (TPP) and AS1411 aptamer structure to realize mitochondria-targeted, CT imaging, and sonodynamic-chemotherapy for cancer treatment (Figure 4A). Relative to the control group, Au-TiO_2_-A-TPP-treated mice displayed significant CT signals at the tumor sites, which validated the capability of Au-TiO_2_-A-TPP to diagnose tumors by CT imaging in vivo (Figure 4B). As the concentration of Au-TiO_2_-A-TPP increased, the brightness and CT values of CT images increased, suggesting a linear relationship between CT grayscale values and concentration (Figure 4C). As mentioned previously, contrast agents commonly used in clinical practice may not be appropriate for patients with renal insufficiency, who may be better suited for contrast agents with a short half-life in vivo and low nephrotoxicity. Surprisingly, the half-life of Au-TiO_2_-A-TPP in the blood circulation of mice was only 4.71 h, indicating Au-TiO_2_-A-TPP was very promising for patients with renal insufficiency (Figure 4D).

Cheng K et al. [48] developed an AgBiS_2_@DSPE-PEG2000-FA (ABS-FA) with good biosafety and active targeted CT imaging capability that combined photothermal and ultrasound kinetic treatment capabilities. It was found that ABS-FA had significant targeting in tumor tissues in vivo, and the CT signals at the site of the tumor were steadily enhanced after ABS-FA (300 μL, 5 mg/mL) injection, reaching a peak at 6–12 h. However, the non-targeted ABS-NH_2_ signals did not change at different time points at the tumor site (Figure 4E). The results of low-power (0.35 W/cm^2^) infrared thermography were consistent with those of CT, which showed that the thermal signal appeared 2 h after drug injection and reached its maximum 10 h after injection (Figure 4F).

Zhang et al. [75] constructed a novel oral nanoparticle, Au@mSiO_2_/Ce6/DOX/SLB-FA@CMC (GMCDS-FA@CMC), that endowed the pH/ultrasonic dual-response to realize the combination of SDT with chemotherapy for colorectal cancer treatment. After oral delivery of GMCDS-FA@CMC, a well-defined tumor CT signal was observed in situ in colorectal cancer model mice and persisted for 7–9 h. It was found that the enteric-coated particles possessed good CT imaging effects in vivo by oral delivery and could be used to direct SDT-chemotherapy for colorectal cancer treatment.

### 5.3. Magnetic Resonance Imaging (MRI)

Since the first implementation of MRI in 1973 as a non-invasive and multi-contrast detection method, MR imaging has been widely used in various biomedical fields [76]. MRI can reflect tissue lesions by combining parameters such as flow effects and electromagnetic wave-related proton density after the excitation of strong magnetic field pulses and the formation of magnetic resonance phenomena through hydrogen atoms in human water molecules [77]. The images are also processed with the aid of computer technology to obtain an excellent diagnosis of the pathology, which has high spatial and tissue resolution [78,79,80]. Therefore, it is extensively utilized clinically in the diagnosis and prognostic status of various diseases. Magnetic resonance imaging is not sensitive, but this obstacle can be overcome by exogenous contrast agents by decreasing the relaxation time of bulk water [80,81]. It was found that appropriate contrast agents are important for enhancing the susceptibility and specificity of diagnosis, enhancing the degree of signal contrast, and improving the resolution of soft tissue images for clinical application [76]. For example, with the help of gadolinium (Gd)-based T_1_ agents, information about the boundaries of brain tumors can be observed more clearly [82]. In recent years, some new nuclear magnetic sensitizers containing Mn and Fe have been applied to MRI tumor imaging [83,84]. Table 3 lists the sonosensitizers and imaging capabilities used for MRI imaging.

**Figure 5:**
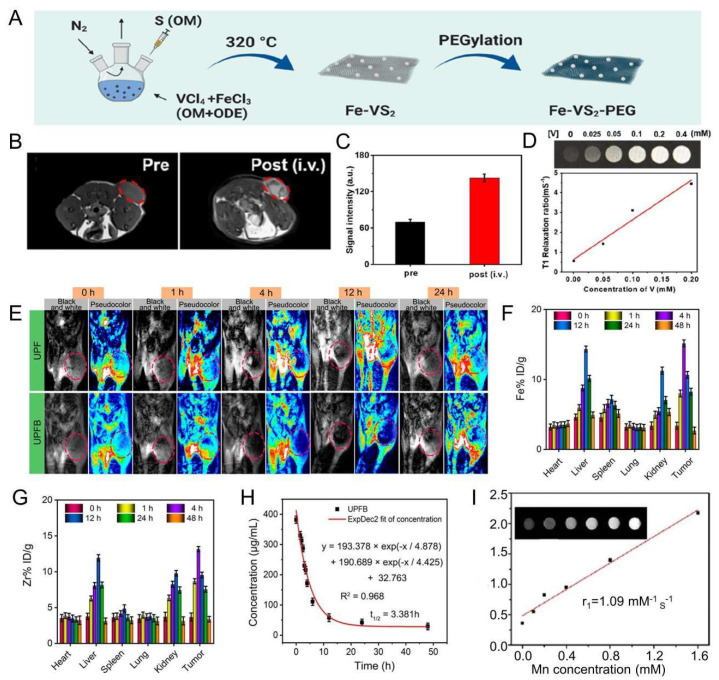
(**A**) Schematic diagram of the synthesis of Fe-VS_2_ NSs. (**B**) Comparative T_1_-weighted MR grayscale images of 4T1 tumor-bearing mice before and after intravenous injection of Fe-VS_2_-PEG NSs. The red circles highlight the tumor sites. (**C**) Qualitative analysis of MR intensity showed in B. (**D**) T_1_-weighted magnetic resonance image and *r*_1_ of Fe-VS_2_-PEG solutions at different molar concentrations of V. (**E**) In vivo T_2_-MRI of tumor-bearing mice by intravenous injection of UPF or UPFB at different time intervals. Mice were imaged on a 1.2 T MRI scanner (Shanghai, China) for different periods. Tumor sites are marked with red ellipses. (**F**) Biodistribution of Zr (% injected dose (ID) of Zr per gram of tissues) in major tissues and tumors at different times after injection of UPFB. (**H**) Fe (% injected dose (ID) of Fe per gram of tissues) content in major tissues and tumors at different times after UPFB injection. (**G**) Blood circulation profile after intravenous injection of UPFB (n = 5). The half-time (t_1/2_) was calculated to be ≈3.381 h. (**I**) T_1_ relaxation rate (1/T_1_) of mZMD NCs measured using the concentration of Mn^2+^ (inset: MR images of different concentrations of mZMD NCs in PBS (pH 6.5). Notes: (**A**–**D**) Adapted with permission from Ref. [83]. Copyright 2020, American Chemical Society. (**E**–**H**) Adapted with permission from Ref. [84]. Copyright 2021, American Chemical Society. (**I**) Adapted with permission from Ref. [91]. Copyright 2022 Wiley-VCH GmbH.

Lei et al. [83] recommended iron-doped vanadium disulfide nanosheets (Fe-VS_2_ NSs) as novel sonosensitizers modified with polyethylene glycol (PEG) to achieve the combination of SDT with chemodynamic therapy (CDT) for cancer therapy. Fe-VS_2_-PEG NSs have magnetic resonance imaging capability and strong tumor inhibility in vivo. At 24 h after intravenous administration of Fe-VS_2_-PEG, MR imaging of the tumor demonstrated significant enhancement, and the quantitative analysis showed that signal strength was 2.04 times stronger than that before administration. (Figure 5B,C). In addition, the concentration of Fe-VS_2_-PEG NSs was positively correlated with MR signal intensity (Figure 5D).

Wang et al. [84] designed and constructed Janus nanostructures called UPFB, consisting of upconversion nanoparticles (UCNPs) (NaYF_4_:20%Yb, 1%Tm@NaYF_4_:10% Yb@NaNdF_4_) and porphyrin-based metal organic frameworks (MOFs) (PCN-224(Fe)). UPFB promoted ROS production by GSH depletion and oxygen supply and realized the integration of SDT and chemodynamic therapy (CDT) for oncology treatment under MRI guidance. Compared with the UPF group, the UPFB group darkened overtime at the tumor site, indicating that the T_2_ contrast signal was enhanced and the accumulation of UPFB was in a time-related manner (Figure 5E). UPFB was highly enriched in tumor tissues by quantitative analysis of the biodistribution of Zr and Fe elements by inductively coupled plasma mass spectrometry (ICP-MS) Figure 5F,G). Furthermore, the circulation half-life of UPFB was calculated to be 3.381 h (Figure 5H). All these results suggested that the UPFB possessed excellent tumor target ability and could be utilized as a contrast agent for T_2_-weighted.

Guan et al. [91] reported a novel biodegradable nanomaterial derived from a mesoporous zeolitic-imidazolate framework@MnO_2_/doxorubicin hydrochloride (ZIF-90@MnO_2_/DOX, mZMD NCs) to achieve T_1_-weighted MRI-guided SDT/CDT/chemotherapy. It was found that the MR image brightness increased with the concentration of mZMD NCs (Figure 5I). After 8 h of intravenous administration, the tumor region was significantly brighter for imaging, indicating that mZMD NCs can effectively aggregate at the tumor site and release Mn^2+^ for T_1_-weighted MR imaging. The mZMD NCs showed good biocompatibility and biosafety and effectively suppressed the growth of tumor cells.

### 5.4. Multi-Modal Imaging

All imaging methods have their disadvantages: MRI has the characteristics of long acquisition time and low space coverage; CT has the risk of ionizing radiation; and the US has limited penetration ability [92,93,94,95]. Single-modality imaging cannot meet the growing demand for accuracy and reliability in clinical diagnostics or clinical research [96]. The combined application of multiple testing techniques has become a hot research topic, complementing each other’s advantages and realizing more precise diseases [97]. Compared with single-mode imaging, multimode imaging achieves multiple imaging functions through a single nanomaterial, providing a basis for accurate cancer diagnosis [98]. To date, several nanoparticle-based bimodal co-imaging materials have been reported to achieve better imaging and treatment.

Wang et al. [99] prepared hollow CoP@N-carbon@PEG (CPCs@PEG) nanospheres (∼60 nm) as sonosensitizers to inhibit tumor growth by promoting ROS production under US irradiation. With the incorporation of cobalt ions, which had magnetic properties and X-ray attenuation coefficients, CPCs@PEG were capable of both CT and MRI. Further, the authors also performed MRI imaging studies in vivo using 4T1 tumor-bearing mice as a model. After the injection of CPC10@PEG, the contrast of the cancer site became darker. In addition, the researchers conducted a CT imaging capability study. With the increase in CPC10@PEG concentration, the CT signal was gradually enhanced. The researchers also investigated the in vivo CT imaging capabilities of CPC10@PEG, which showed a significant increase in the brightness of the cancer site after intravenous injection compared with pre-injection.

Gong et al. [44] designed and prepared a novel high-performance multifunctional sonosensitizer built on ultramicroscopic oxygen-deficient bimetallic oxide MnWO_X_ nanoparticles for multimodal imaging-guided SDT for cancer therapy. The MnWO_X_-PEG nanoparticles exhibited effective SDT effects by producing ^1^O_2_ and ·OH and possessed the glutathione depletion capability to enhance the SDT efficacy. MnWO_X_-PEG exhibited good biosafety and excellent tumor growth suppression in mice under ultrasound irradiation. Due to the high attenuation of X-rays by the W element, MnWO_X_-PEG can also be applied in CT imaging and as a reduction agent for T_1_ in magnetic resonance imaging. The findings indicated that after 24 h of intravenous injection of MnWO_X_-PEG 4T1, the tumor-bearing mice showed significant CT (2.4 times) and MRI (1.8 times) signals in the tumor site. These multimodal imaging results demonstrated that MnWO_X_-PEG can efficiently accumulate in tumors, and sonosensitizers had diagnostic imaging capabilities and assisted in the precise treatment of tumors with SDT.

## 6. Conclusions and Outlook

This review summarized the mechanism of action of SDT and further described the advantages and disadvantages of the three molecular imaging modalities commonly practiced in clinical practice, as well as summarized studies using sonosensitizers as contrast agents. SDT has the advantages of a good therapeutic effect, profound organization penetration, and small collateral damage, which makes it a promising cancer treatment. There have been efforts to use SDT for the treatment of cancer and the production of related sonosensitizers since its discovery in 1989. Now, the door seems to have been opened to the great value of sonosensitizers for imaging in addition to cancer treatment, which is becoming an attractive trend. Clinical imaging techniques contribute to earlier and more predictable detection of cancer, provide an accurate and effective evaluation of tumor treatment, and help patients improve their prognosis and survival. However, the limitations of current clinical contrast agents are taken into account. Therefore, it is particularly important to create sonosensitizers with better diagnostic contrast capabilities. 

First, although current research on sonosensitizers is in full swing, most of them are facing different problems. Feng et al. [8] developed a sequential strategy for ultrasound-mediated nano-therapy based on TPZ/HMTNPs-SNO. When encountered with ultrasound, TPZ/HMTNPs-SNO further sensitizes the release of nitric oxide (NO) bubbles for ultrasound cancer imaging in a controlled manner. However, when compared with clinical ultrasound contrast agents (Albunex, Echovist, etc.), the ultrasound imaging performance of HMTNPs-SNO is not satisfactory due to the limited NO concentration. Although research on sonosensitizers is in full swing, it still faces great challenges, such as low sensitization efficiency, a lack of accumulation in lesion areas, and insufficient research on therapy mechanisms. To overcome these restrictions, cross-disciplinary collaboration shall be strengthened to construct functionalized sonosensitizers with highly productive diagnostic and therapeutic methods.

Second, the biosafety assessment of sonosensitizers is a central barrier to promoting clinical translation. However, the phototoxicity of organic sonosensitizers and the composition of inorganic sonosensitizers greatly limit their clinical translation. Most of the sonosensitizers currently used for CT and MRI contain heavy metals (e.g., manganese and titanium) whose metabolism, biodegradation, bioavailability, and toxicity in humans are unknown. Therefore, we still need to explore sonosensitizers that are less toxic or even non-toxic to humans. Meanwhile, a comprehensive and systematic evaluation of their toxicity and pharmacology is needed to promote the development of sonosensitizers.

Third, from a clinical perspective, many factors remain to be considered to maximize the imaging quality of sonosensitizers. The identification and validation of novel imaging targets must be attended to, and other features must be investigated in the context of the need for clinical application. We hypothesized that we could contribute to the scientific community’s great progress in cancer imaging and therapy by highlighting the latest achievements in this field. The future development of sonosensitizers for SDT will provide valuable information and insights to facilitate the clinical translation of sonosensitizers for SDT for therapeutic and diagnostic purposes. Based on the research advances in this study, these nanoparticles for imaging and therapeutic versatility will hopefully provide new expectations for clinical applications in cancer therapy.

## Figures and Tables

**Table 1:** US Imaging Characteristics of the Multifunctional Sonosensitizers.

Sonosensitizers	Probes	Biological Model	SDT Result	Imaging Effect	Ref.
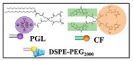 PCF-MBs	PCF	HT-29 cancer-bearing Balb/c nude mice	tumor inhibition rate of more than 50%	20 s post-injection, the US imaging signal reached the maximum; and the contrast enhancement could last for more than 3 min	[49]
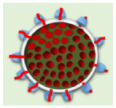 FMSN-DOX	FMSNs	TRAMP tumor-bearing nude mice	The gradual reduction in tumor growth	from day 1 to day 9 with significant contrast enhancement within the tumor.	[45]
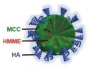 HMME/MCC-HA	MCC NPs	MCF-7 tumor-bearing nude mice	successfully suppressed the tumor volume with the V/V0 of 0.87 ± 0.13	strong US signals in tumor site at 3 h post-injection, and particularly after exposure to US stimulus	[8]
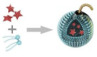 Lip-AIPH	AIPH	MCF-7 tumor-bearing mice	a highly significant antitumor effect was achieved in mice in the group of Lip-AIPH with US irradiation	a highly significant antitumor effect was achieved in mice in the group of Lip-AIPH with US irradiation	[62]
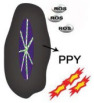 mTiO_2_@PPY-HNK	mTiO_2_	4T1 tumor model	significantly inhibit the tumor growth	as the concentration increases, the ultrasound signal is more intense and the image is clearer	[63]
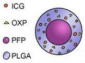 OIX_NPs	PFP	ID8 cells into the left shoulder (the primary tumor)	significant inhibition of tumor volume	peaking at 4 h post-injection.	[64]
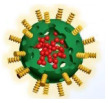 TPZ/HMTNPs-SNO	HMTNPs-SNO	MCF-7 tumor-bearing nude mice	exhibited an effective therapeutic effect	compared with the saline group, showed local enhancement at the tumor site.	[65]
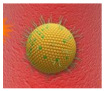 IR780-NDs	PFP	breast cancer 4T1 nude mice	Tumor weight drop	24 h after the injection of IR780-NDs a bright US signal occurred at the tumor site.	[47]
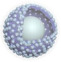 SMISO NPs	SMISO	4T1 tumor-bearing nude mice	the inhibition rate of tumor growth in the SMISO + US group reached 88.2%	the grayscale values of US images increase with them. concentration increases	[66]
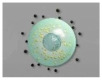 RPPs&SPIOs	RPPs	4T1 tumors	a 100% survival rate of mice at 90 days	Shift in RPPs after thermal stimulation results in significant contrast enhancement	[67]
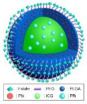 FA-PEG-PLGA-Ptx@ICG-Pfh NPs	Pfh	MDA-MB231 tumor-bearing mice	tumor growth was significantly inhibited	images were greatly improved	[68]
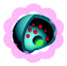 RBC-HPBs/HMME/PFH	PFH	4T1 tumor-bearing female mice	enhancing tumor treatment effects of HMME	A clear US signal was observed at 4 h after injection, and the strongest signal appeared at 8 h.	[69]
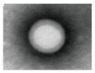 Ce6-PFP-DTX/PLGA	PFP	breast cancer 4T1 nude mice	much higher inhibition rate of the CPDP NPs + LIFU group	after LIFU irradiation, the corresponding intensity of CPDP NPs was elevated compared with the pre-irradiation group	[70]
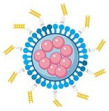 AS1411-DOX-PFH-PEG@PLGA	PFH	breast cancer 4T1 nude mice	the tumor volumes significantly decreased	Increased imaging ability of ADPPs in vivo within 24 h after intravenous injection	[71]
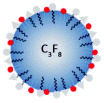 RB-MBs	MBs	HT-29 tumor mouse model	-	a contrast-enhanced ultrasound imaging mode at a frequency of 5 MHz	[72]

**Table 2:** CT Imaging Characteristics of the Multifunctional Sonosensitizers.

Sonosensitizers	Probes	Biological Model	Treatment Result	Imaging Effect	Ref.
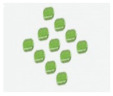 MnWO_X_-PEG	W(CO)_6_	4T1 tumor-bearing mice	Tumor weight drop	CT imaging signal intensity was almost 2.4 times higher than that of the control group	[44]
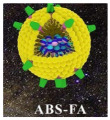 AgBiS_2_@DSPE-PEG_2000_-FA	AgBiS_2_	HeLa tumor-bearing mice	Tumor size drop	The CT signal intensity at the tumor site gradually increased and peaked at 6h after the injection	[48]
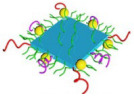 Au-TiO_2_-A-TPP	Au-TiO_2_	MCF-7 tumor-bearing mice	Tumor weight drop	The CT signal in the tumor area reached its maximum at 24 h	[51]
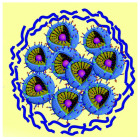 GMCDS-FA@CMC	Au@mSiO_2_	orthotopic colorectal tumor	Decreased number and smaller diameter of colorectal tumors	The nanoprobe remained in the colorectal region	[75]

**Table 3:** MR Imaging Characteristics of the Multifunctional Sonosensitizers.

Sonosensitizers	Probes	Biological Model	SDT Result	Imaging Effect	Ref.
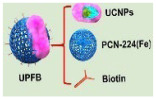 UPFB	MOFs (Fe^3+^)	U14 tumor-bearing Kunming mice	increased inhibition of tumor growth	enhanced T_2_ contrast signal	[84]
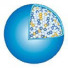 Fe-TiO_2_ NDs	Fe^3+^	4T1 tumor-bearing mice	better tumor inhibition	The brightening effect through the T_1_-weighted MR images	[85]
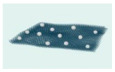 Fe-VS_2_-PEG	Fe^3+^	4T1 cells subcutaneously in BALB/c mice	better treatment effect and a longer survival period	the tumor had an obvious brightening effect 24 h after i.v. injection (T_1_)	[83]
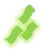 OCN-PEG-(Ce6-Gd^3+^)/BNN6	Ga^3+^	4T1 tumor-bearing mice	the tumor suppression rate reached 63.2%	The T_1_-weighted contrast effect was significantly enhanced	[52]
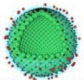 MnSiO_3_-Pt@BSA-Ce6(MPBC)	Mn^2+^	U14 tumor-bearing Balb/c mice	tumor growth inhibition	the T1−MR signal of the tumor enhanced	[86]
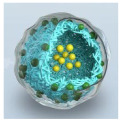 MG@P NPs	PMnC (Mn)	4T1 tumor-bearing mice	the tumor growth of Mice remarkably suppressed	T_1_ signal of the tumor region showed an increasing trend within 24 h and then decreased	[87]
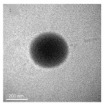 F3-PLGA@MB/Gd NPs	Gd-DTPA-BMA	the nude mice bearing MDA-MB-231 tumors	induce tumor cell apoptosis	Linear signal dependence of T_1_ intensity values	[46]
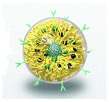 Ang-IR780-MnO_2_-PLGA (AIMP)	MnO_2_	U87MG tumor-bearing mice	enhanced SDT effect	A linear relationship was shown between the 1/T_1_ values and Mn concentration	[88]
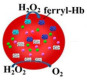 DOX/Mn-TPPS@RBCS	Mn-TPPS	MCF-7 tumor-bearing nude mice	Inhibit tumor growth	T_1_-weighted MR imaging results in enhancement	[89]
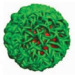 MnTTP-HSA	MnTTP (Mn)	MCF-7 tumor-bearing nude mice	the best in completely inhibiting tumor	a T_1_ the positive signal at the tumor showed an increasing trend within 3 h and then gradually decreased	[90]
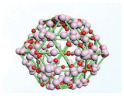 mZMD	MnO_2_	HeLa tumor xenograft-bearing nude mice	significant suppression effects	the concentration of the NCs increased, the T_1_ MR images became brighter and brighter	[91]
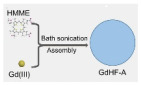 GdHF-NDs	Ga^3+^	CT26 tumor-bearing mice	GdHF-NDs/PEG + US shows the most potent efficiency in tumor suppression (73.7%)	T_1_ signal strength increased	[78]

## Data Availability

Not applicable.

## Sample Availability

Samples of the compounds are not available from the authors.

## Abbreviations

SDT, Sonodynamic therapy; USI, Ultrasound imaging; CT, X-ray computed tomography; MRI, Magnetic resonance imaging; PTT, Photothermal therapy; PDT, Photodynamic therapy; PTAs, Photothermal agents; ROS Reactive oxygen species; O_2_^−^, Superoxides; H_2_O_2_, Hydrogen peroxide; ·OH, Hydroxyl radicals; Cyt c, Cytochrome c; DCs, Dendritic cells; HIFU, High-intensity focused US; UV, Ultraviolet; NPs, Nanoparticles; PAI, Photoacoustic imaging; FLI, Fluorescence imaging; CA, Contrast agents; US, Ultrasound; CEUS, Contrast-enhanced ultrasound; nIR, near infra-red; MBs, Microbubbles; NBs, Nanobubbles; PFP, Perfluoropentane; INBs, Interfacial nanobubbles; PCF-MBs, Porphyrin/camptothecin-floxuridine triad microbubbles; TiO_2_, Titanium oxide; TPP, Triphenylphosphine; MOFs, Metal organic frameworks; CDT, Chemodynamic therapy; NO, Nitric oxide; ICP-MS, Inductively coupled plasma mass spectrometry.

## References

[1] Sung H., Ferlay J., Siegel R.L., Laversanne M., Soerjomataram I., Jemal A., Bray F. (2021). Global Cancer Statistics 2020: GLOBOCAN Estimates of Incidence and Mortality Worldwide for 36 Cancers in 185 Countries. CA Cancer J. Clin..

[2] Wu N., Fan C.H., Yeh C.K. (2022). Ultrasound-activated nanomaterials for sonodynamic cancer theranostics. Drug Discov. Today.

[3] Pan X., Wang H., Wang S., Sun X., Wang L., Wang W., Shen H., Liu H. (2018). Sonodynamic therapy (SDT): A novel strategy for cancer nanotheranostics. Sci. China Life Sci..

[4] Gordon Spratt E.A., Gorcey L.V., Soter N.A., Brauer J.A. (2015). Phototherapy, photodynamic therapy and photophoresis in the treatment of connective-tissue diseases: A review. Br. J. Dermatol..

[5] Yu J., Chu C., Wu Y., Liu G., Li W. (2021). The phototherapy toward corneal neovascularization elimination: An efficient, selective and safe strategy. Chin. Chem. Lett..

[6] Zhao L., Zhang X., Wang X., Guan X., Zhang W., Ma J. (2021). Recent advances in selective photothermal therapy of tumor. J. Nanobiotechnol..

[7] Shang T., Yu X., Han S., Yang B. (2020). Nanomedicine-based tumor photothermal therapy synergized immunotherapy. Biomater. Sci..

[8] Feng Q., Zhang W., Yang X., Li Y., Hao Y., Zhang H., Hou L., Zhang Z. (2018). pH/Ultrasound Dual-Responsive Gas Generator for Ultrasound Imaging-Guided Therapeutic Inertial Cavitation and Sonodynamic Therapy. Adv. Healthc. Mater..

[9] Czarnecka-Czapczyńska M., Aebisher D., Oleś P., Sosna B., Krupka-Olek M., Dynarowicz K., Latos W., Cieślar G., Kawczyk-Krupka A. (2021). The role of photodynamic therapy in breast cancer—A review of in vitro research. Biomed. Pharmacother. Biomed. Pharmacother..

[10] Liao S., Cai M., Zhu R., Fu T., Du Y., Kong J., Zhang Y., Qu C., Dong X., Ni J. (2023). Antitumor Effect of Photodynamic Therapy/Sonodynamic Therapy/Sono-Photodynamic Therapy of Chlorin e6 and Other Applications. Mol. Pharm..

[11] Yang Y., Huang J., Liu M., Qiu Y., Chen Q., Zhao T., Xiao Z., Yang Y., Jiang Y., Huang Q. (2023). Emerging Sonodynamic Therapy-Based Nanomedicines for Cancer Immunotherapy. Adv. Sci..

[12] Zha B., Yang J., Dang Q., Li P., Shi S., Wu J., Cui H., Huangfu L., Li Y., Yang D. (2023). A phase I clinical trial of sonodynamic therapy combined with temozolomide in the treatment of recurrent glioblastoma. J. Neuro-Oncol..

[13] Huang S., Ding D., Lan T., He G., Ren J., Liang R., Zhong H., Chen G., Lu X., Shuai X. (2023). Multifunctional nanodrug performs sonodynamic therapy and inhibits TGF-β to boost immune response against colorectal cancer and liver metastasis. Acta Biomater..

[14] Brault D. (1990). Physical chemistry of porphyrins and their interactions with membranes: The importance of pH. J. Photochem. Photobiol. B Biol..

[15] Vrouenraets M.B., Visser G.W., Snow G.B., van Dongen G.A. (2003). Basic principles, applications in oncology and improved selectivity of photodynamic therapy. Anticancer Res..

[16] Sofuni A., Itoi T. (2022). Current status and future perspective of sonodynamic therapy for cancer. J. Med. Ultrason..

[17] Yumita N., Nishigaki R., Umemura K., Umemura S. (1989). Hematoporphyrin as a sensitizer of cell-damaging effect of ultrasound. Jpn. J. Cancer Res. Gann.

[18] Umemura S., Kawabata K., Yurnita N., Nishigaki R., Umemura K. Sonodynamic approach to tumor treatment. Proceedings of the IEEE 1992 Ultrasonics Symposium Proceedings.

[19] Li D., Yang Y., Li D., Pan J., Chu C., Liu G. (2021). Organic Sonosensitizers for Sonodynamic Therapy: From Small Molecules and Nanoparticles toward Clinical Development. Small.

[20] Hong L., Pliss A.M., Zhan Y., Zheng W., Xia J., Liu L., Qu J., Prasad P.N. (2020). Perfluoropolyether Nanoemulsion Encapsulating Chlorin e6 for Sonodynamic and Photodynamic Therapy of Hypoxic Tumor. Nanomaterials.

[21] Yumita N., Iwase Y., Nishi K., Ikeda T., Komatsu H., Fukai T., Onodera K., Nishi H., Takeda K., Umemura S. (2011). Sonodynamically-induced antitumor effect of mono-l-aspartyl chlorin e6 (NPe6). Anticancer Res..

[22] Wan G.Y., Liu Y., Chen B.W., Liu Y.Y., Wang Y.S., Zhang N. (2016). Recent advances of sonodynamic therapy in cancer treatment. Cancer Biol. Med..

[23] Honda H., Kondo T., Zhao Q.L., Feril L.B., Kitagawa H. (2004). Role of intracellular calcium ions and reactive oxygen species in apoptosis induced by ultrasound. Ultrasound Med. Biol..

[24] Gao Z., Zheng J., Yang B., Wang Z., Fan H., Lv Y., Li H., Jia L., Cao W. (2013). Sonodynamic therapy inhibits angiogenesis and tumor growth in a xenograft mouse model. Cancer Lett..

[25] Wang S., Hu Z., Wang X., Gu C., Gao Z., Cao W., Zheng J. (2014). 5-Aminolevulinic acid-mediated sonodynamic therapy reverses macrophage and dendritic cell passivity in murine melanoma xenografts. Ultrasound Med. Biol..

[26] Liu F., Hu Z., Qiu L., Hui C., Li C., Zhong P., Zhang J. (2010). Boosting high-intensity focused ultrasound-induced anti-tumor immunity using a sparse-scan strategy that can more effectively promote dendritic cell maturation. J. Transl. Med..

[27] Kennedy J.E. (2005). High-intensity focused ultrasound in the treatment of solid tumours. Nat. Rev. Cancer.

[28] Williams A.R. (1972). Disorganization and Disruption of Mammalian and Amoeboid Cells by Acoustic Microstreaming. J. Acoust. Soc. Am..

[29] Haar G.T., Coussios C. (2007). High intensity focused ultrasound: Physical principles and devices. Int. J. Hyperth..

[30] Lauterborn W., Kurz T., Geisler R., Schanz D., Lindau O. (2007). Acoustic cavitation, bubble dynamics and sonoluminescence. Ultrason. Sonochem..

[31] Rengeng L., Qianyu Z., Yuehong L., Zhongzhong P., Libo L. (2017). Sonodynamic therapy, a treatment developing from photodynamic therapy. Photodiagnosis Photodyn. Ther..

[32] Pecha R., Gompf B. (2000). Microimplosions: Cavitation collapse and shock wave emission on a nanosecond time scale. Phys. Rev. Lett..

[33] Leighton T. (2012). The Acoustic Bubble.

[34] Rooney J.A. (1970). Hemolysis near an ultrasonically pulsating gas bubble. Science.

[35] Rosenthal I., Sostaric J.Z., Riesz P. (2004). Sonodynamic therapy--a review of the synergistic effects of drugs and ultrasound. Ultrason. Sonochemistry.

[36] Yildirim A., Chattaraj R., Blum N.T., Goldscheitter G.M., Goodwin A.P. (2016). Stable Encapsulation of Air in Mesoporous Silica Nanoparticles: Fluorocarbon-Free Nanoscale Ultrasound Contrast Agents. Adv. Healthc. Mater..

[37] Brazzale C., Canaparo R., Racca L., Foglietta F., Durando G., Fantozzi R., Caliceti P., Salmaso S., Serpe L. (2016). Enhanced selective sonosensitizing efficacy of ultrasound-based anticancer treatment by targeted gold nanoparticles. Nanomedicine.

[38] Suslick K.S., Doktycz S.J., Flint E.B. (1990). On the origin of sonoluminescence and sonochemistry. Ultrasonics.

[39] Yin H., Chang N., Xu S., Wan M. (2016). Sonoluminescence characterization of inertial cavitation inside a BSA phantom treated by pulsed HIFU. Ultrason. Sonochem..

[40] Kessel D., Jeffers R., Fowlkes J.B., Cain C. (1994). Porphyrin-induced enhancement of ultrasound cytotoxicity. Int. J. Radiat. Biol..

[41] Misík V., Riesz P. (2000). Free radical intermediates in sonodynamic therapy. Ann. N. Y. Acad. Sci..

[42] Warner S. (2004). Diagnostics + therapy = theranostics: Strategy requires teamwork, partnering, and tricky regulatory maneuvering. Scientist.

[43] Ke H., Wang J., Dai Z., Jin Y., Qu E., Xing Z., Guo C., Yue X., Liu J. (2011). Gold-nanoshelled microcapsules: A theranostic agent for ultrasound contrast imaging and photothermal therapy. Angew. Chem..

[44] Gong F., Cheng L., Yang N., Betzer O., Feng L., Zhou Q., Li Y., Chen R., Popovtzer R., Liu Z. (2019). Ultrasmall Oxygen-Deficient Bimetallic Oxide MnWO_X_ Nanoparticles for Depletion of Endogenous GSH and Enhanced Sonodynamic Cancer Therapy. Adv. Mater..

[45] Ho Y.J., Wu C.H., Jin Q.F., Lin C.Y., Chiang P.H., Wu N., Fan C.H., Yang C.M., Yeh C.K. (2020). Superhydrophobic drug-loaded mesoporous silica nanoparticles capped with β-cyclodextrin for ultrasound image-guided combined antivascular and chemo-sonodynamic therapy. Biomaterials.

[46] Li Y., Hao L., Liu F., Yin L., Yan S., Zhao H., Ding X., Guo Y., Cao Y., Li P. (2019). Cell penetrating peptide-modified nanoparticles for tumor targeted imaging and synergistic effect of sonodynamic/HIFU therapy. Int. J. Nanomed..

[47] Zhang L., Yi H., Song J., Huang J., Yang K., Tan B., Wang D., Yang N., Wang Z., Li X. (2019). Mitochondria-Targeted and Ultrasound-Activated Nanodroplets for Enhanced Deep-Penetration Sonodynamic Cancer Therapy. ACS Appl. Mater. Interfaces.

[48] Cheng K., Zhang R.Y., Yang X.Q., Zhang X.S., Zhang F., An J., Wang Z.Y., Dong Y., Liu B., Zhao Y.D. (2020). One-for-All Nanoplatform for Synergistic Mild Cascade-Potentiated Ultrasound Therapy Induced with Targeting Imaging-Guided Photothermal Therapy. ACS Appl. Mater. Interfaces.

[49] Chen M., Liang X., Gao C., Zhao R., Zhang N., Wang S., Chen W., Zhao B., Wang J., Dai Z. (2018). Ultrasound Triggered Conversion of Porphyrin/Camptothecin-Fluoroxyuridine Triad Microbubbles into Nanoparticles Overcomes Multidrug Resistance in Colorectal Cancer. ACS Nano.

[50] Nomikou N., Curtis K., McEwan C., O’Hagan B.M.G., Callan B., Callan J.F., McHale A.P. (2017). A versatile, stimulus-responsive nanoparticle-based platform for use in both sonodynamic and photodynamic cancer therapy. Acta Biomater..

[51] Cao Y., Wu T., Dai W., Dong H., Zhang X. (2019). TiO_2_ Nanosheets with the Au Nanocrystal-Decorated Edge for Mitochondria-Targeting Enhanced Sonodynamic Therapy. Chem. Mater..

[52] Zheng Y., Liu Y., Wei F., Xiao H., Mou J., Wu H., Yang S. (2021). Functionalized g-C_3_N_4_ nanosheets for potential use in magnetic resonance imaging-guided sonodynamic and nitric oxide combination therapy. Acta Biomater..

[53] Wang C., Tian Y., Wu B., Cheng W. (2022). Recent Progress Toward Imaging Application of Multifunction Sonosensitizers in Sonodynamic Therapy. Int. J. Nanomed..

[54] Xu C., Huang J., Jiang Y., He S., Zhang C., Pu K. (2023). Nanoparticles with ultrasound-induced afterglow luminescence for tumour-specific theranostics. Nat. Biomed. Eng..

[55] Dong C., Jiang Q., Qian X., Wu W., Wang W., Yu L., Chen Y. (2020). A self-assembled carrier-free nanosonosensitizer for photoacoustic imaging-guided synergistic chemo-sonodynamic cancer therapy. Nanoscale.

[56] Slagle C.J., Thamm D.H., Randall E.K., Borden M.A. (2018). Click Conjugation of Cloaked Peptide Ligands to Microbubbles. Bioconjugate Chem..

[57] Wang Y., Cong H., Wang S., Yu B., Shen Y. (2021). Development and application of ultrasound contrast agents in biomedicine. J. Mater. Chem. B.

[58] Kloth C., Kratzer W., Schmidberger J., Beer M., Clevert D.A., Graeter T. (2021). Ultrasound 2020—Diagnostics & Therapy: On the Way to Multimodal Ultrasound: Contrast-Enhanced Ultrasound (CEUS), Microvascular Doppler Techniques, Fusion Imaging, Sonoelastography, Interventional Sonography. RöFo-Fortschritte auf dem Gebiet der Röntgenstrahlen und der Bildgebenden Verfahren.

[59] Claudon M., Dietrich C.F., Choi B.I., Cosgrove D.O., Kudo M., Nolsøe C.P., Piscaglia F., Wilson S.R., Barr R.G., Chammas M.C. (2013). Guidelines and good clinical practice recommendations for Contrast Enhanced Ultrasound (CEUS) in the liver—Update 2012: A WFUMB-EFSUMB initiative in cooperation with representatives of AFSUMB, AIUM, ASUM, FLAUS and ICUS. Ultrasound Med. Biol..

[60] Huynh E., Rajora M.A., Zheng G. (2016). Multimodal micro, nano, and size conversion ultrasound agents for imaging and therapy. Wiley Interdiscip. Rev. Nanomed. Nanobiotechnol..

[61] Sun S., Xu Y., Fu P., Chen M., Sun S., Zhao R., Wang J., Liang X., Wang S. (2018). Ultrasound-targeted photodynamic and gene dual therapy for effectively inhibiting triple negative breast cancer by cationic porphyrin lipid microbubbles loaded with HIF1α-siRNA. Nanoscale.

[62] Lin X., Qiu Y., Song L., Chen S., Chen X., Huang G., Song J., Chen X., Yang H. (2019). Ultrasound activation of liposomes for enhanced ultrasound imaging and synergistic gas and sonodynamic cancer therapy. Nanoscale Horiz..

[63] He Y., Wan J., Yang Y., Yuan P., Yang C., Wang Z., Zhang L. (2019). Multifunctional Polypyrrole-Coated Mesoporous TiO_2_ Nanocomposites for Photothermal, Sonodynamic, and Chemotherapeutic Treatments and Dual-Modal Ultrasound/Photoacoustic Imaging of Tumors. Adv. Healthc. Mater..

[64] Zheng J., Sun J., Chen J., Zhu S., Chen S., Liu Y., Hao L., Wang Z., Chang S. (2021). Oxygen and oxaliplatin-loaded nanoparticles combined with photo-sonodynamic inducing enhanced immunogenic cell death in syngeneic mouse models of ovarian cancer. J. Control. Release.

[65] Feng Q., Li Y., Yang X., Zhang W., Hao Y., Zhang H., Hou L., Zhang Z. (2018). Hypoxia-specific therapeutic agents delivery nanotheranostics: A sequential strategy for ultrasound mediated on-demand tritherapies and imaging of cancer. J. Control. Release.

[66] Zhang T., Zheng Q., Xie C., Fan G., Wang Y., Wu Y., Fu Y., Huang J., Craig D.Q.M., Cai X. (2023). Integration of Silica Nanorattles with Manganese-Doped In_2_S_3_/InOOH to Enable Ultrasound-Mediated Tumor Theranostics. ACS Appl. Mater. Interfaces.

[67] Qin Q., Zhou Y., Li P., Liu Y., Deng R., Tang R., Wu N., Wan L., Ye M., Zhou H. (2023). Phase-transition nanodroplets with immunomodulatory capabilities for potentiating mild magnetic hyperthermia to inhibit tumour proliferation and metastasis. J. Nanobiotechnol..

[68] Liu F., Chen Y., Li Y., Guo Y., Cao Y., Li P., Wang Z., Gong Y., Ran H. (2018). Folate-receptor-targeted laser-activable poly(lactide-*co*-glycolic acid) nanoparticles loaded with paclitaxel/indocyanine green for photoacoustic/ultrasound imaging and chemo/photothermal therapy. Int. J. Nanomed..

[69] Zhang H., Chen J., Zhu X., Ren Y., Cao F., Zhu L., Hou L., Zhang H., Zhang Z. (2018). Ultrasound induced phase-transition and invisible nanobomb for imaging-guided tumor sonodynamic therapy. J. Mater. Chem. B.

[70] Zhang Q., Wang W., Shen H., Tao H., Wu Y., Ma L., Yang G., Chang R., Wang J., Zhang H. (2021). Low-Intensity Focused Ultrasound-Augmented Multifunctional Nanoparticles for Integrating Ultrasound Imaging and Synergistic Therapy of Metastatic Breast Cancer. Nanoscale Res. Lett..

[71] Kang Z., Yang M., Feng X., Liao H., Zhang Z., Du Y. (2022). Multifunctional Theranostic Nanoparticles for Enhanced Tumor Targeted Imaging and Synergistic FUS/Chemotherapy on Murine 4T1 Breast Cancer Cell. Int. J. Nanomed..

[72] Hou R., Liang X., Li X., Zhang X., Ma X., Wang F. (2020). In situ conversion of rose bengal microbubbles into nanoparticles for ultrasound imaging guided sonodynamic therapy with enhanced antitumor efficacy. Biomater. Sci..

[73] Jiang Z., Zhang M., Li P., Wang Y., Fu Q. (2023). Nanomaterial-based CT contrast agents and their applications in image-guided therapy. Theranostics.

[74] Pan X., Siewerdsen J., La Riviere P.J., Kalender W.A. (2008). Anniversary paper. Development of x-ray computed tomography: The role of medical physics and AAPM from the 1970s to present. Med. Phys..

[75] Zhang R.Y., Cheng K., Xuan Y., Yang X.Q., An J., Hu Y.G., Liu B., Zhao Y.D. (2021). A pH/ultrasonic dual-response step-targeting enterosoluble granule for combined sonodynamic-chemotherapy guided via gastrointestinal tract imaging in orthotopic colorectal cancer. Nanoscale.

[76] Guo L., Xi J., Teng J., Wang J., Chen Y. (2022). Magnetic Resonance Neuroimaging Contrast Agents of Nanomaterials. Biomed. Res. Int..

[77] Yuan P., Song D. (2018). MRI tracing non-invasive TiO_2_-based nanoparticles activated by ultrasound for multi-mechanism therapy of prostatic cancer. Nanotechnology.

[78] Geng P., Yu N., Liu X., Zhu Q., Wen M., Ren Q., Qiu P., Zhang H., Li M., Chen Z. (2021). Sub 5 nm Gd^3+^-Hemoporfin Framework Nanodots for Augmented Sonodynamic Theranostics and Fast Renal Clearance. Adv. Healthc. Mater..

[79] Geethanath S., Vaughan J.T. (2019). Accessible magnetic resonance imaging: A review. J. Magn. Reson. Imaging.

[80] Pellico J., Ellis C.M., Davis J.J. (2019). Nanoparticle-Based Paramagnetic Contrast Agents for Magnetic Resonance Imaging. Contrast Media Mol. Imaging.

[81] De León-Rodríguez L.M., Martins A.F., Pinho M.C., Rofsky N.M., Sherry A.D. (2015). Basic MR relaxation mechanisms and contrast agent design. J. Magn. Reson. Imaging.

[82] Abd-Ellah M.K., Awad A.I., Khalaf A.A.M., Hamed H.F.A. (2019). A review on brain tumor diagnosis from MRI images: Practical implications, key achievements, and lessons learned. Magn. Reson. Imaging.

[83] Lei H., Wang X., Bai S., Gong F., Yang N., Gong Y., Hou L., Cao M., Liu Z., Cheng L. (2020). Biodegradable Fe-Doped Vanadium Disulfide Theranostic Nanosheets for Enhanced Sonodynamic/Chemodynamic Therapy. ACS Appl. Mater. Interfaces.

[84] Wang Z., Liu B., Sun Q., Feng L., He F., Yang P., Gai S., Quan Z., Lin J. (2021). Upconverted Metal-Organic Framework Janus Architecture for Near-Infrared and Ultrasound Co-Enhanced High Performance Tumor Therapy. ACS Nano.

[85] Bai S., Yang N., Wang X., Gong F., Dong Z., Gong Y., Liu Z., Cheng L. (2020). Ultrasmall Iron-Doped Titanium Oxide Nanodots for Enhanced Sonodynamic and Chemodynamic Cancer Therapy. ACS Nano.

[86] Jiang F., Yang C., Ding B., Liang S., Zhao Y., Cheng Z., Liu M., Xing B., Ma P., Lin J. (2022). Tumor microenvironment-responsive MnSiO_3_-Pt@BSA-Ce6 nanoplatform for synergistic catalysis-enhanced sonodynamic and chemodynamic cancer therapy. Chin. Chem. Lett..

[87] Wang J., Huang J., Zhou W., Zhao J., Peng Q., Zhang L., Wang Z., Li P., Li R. (2021). Hypoxia modulation by dual-drug nanoparticles for enhanced synergistic sonodynamic and starvation therapy. J. Nanobiotechnol..

[88] Liu S., Zhang W., Chen Q., Hou J., Wang J., Zhong Y., Wang X., Jiang W., Ran H., Guo D. (2021). Multifunctional nanozyme for multimodal imaging-guided enhanced sonodynamic therapy by regulating the tumor microenvironment. Nanoscale.

[89] Du B., Yan X., Ding X., Wang Q., Du Q., Xu T., Shen G., Yao H., Zhou J. (2018). Oxygen Self-Production Red Blood Cell Carrier System for MRI Mediated Cancer Therapy: Ferryl-Hb, Sonodynamic, and Chemical Therapy. ACS Biomater. Sci. Eng..

[90] Wang L., Song W., Choi S., Yu K., Zhang F., Guo W., Ma Y., Wang K., Qu F., Lin H. (2023). Hollow CoP@N-Carbon Nanospheres: Heterostructure and Glucose-Enhanced Charge Separation for Sonodynamic/Starvation Therapy. ACS Appl. Mater. Interfaces.

[91] Ma A., Chen H., Cui Y., Luo Z., Liang R., Wu Z., Chen Z., Yin T., Ni J., Zheng M. (2019). Metalloporphyrin Complex-Based Nanosonosensitizers for Deep-Tissue Tumor Theranostics by Noninvasive Sonodynamic Therapy. Small.

[92] Guan S., Liu X., Li C., Wang X., Cao D., Wang J., Lin L., Lu J., Deng G., Hu J. (2022). Intracellular Mutual Amplification of Oxidative Stress and Inhibition Multidrug Resistance for Enhanced Sonodynamic/Chemodynamic/Chemo Therapy. Small.

[93] Neuschmelting V., Harmsen S., Beziere N., Lockau H., Hsu H.T., Huang R., Razansky D., Ntziachristos V., Kircher M.F. (2018). Dual-Modality Surface-Enhanced Resonance Raman Scattering and Multispectral Optoacoustic Tomography Nanoparticle Approach for Brain Tumor Delineation. Small.

[94] Salvatori M., Rizzo A., Rovera G., Indovina L., Schillaci O. (2019). Radiation dose in nuclear medicine: The hybrid imaging. Radiol. Med..

[95] Perry J.L., Mason K., Sutton B.P., Kuehn D.P. (2018). Can Dynamic MRI Be Used to Accurately Identify Velopharyngeal Closure Patterns?. Cleft Palate-Craniofacial J..

[96] Guo W., Chen Z., Tan L., Gu D., Ren X., Fu C., Wu Q., Meng X. (2021). Emerging biocompatible nanoplatforms for the potential application in diagnosis and therapy of deep tumors. View.

[97] Jennings L.E., Long N.J. (2009). ‘Two is better than one’--probes for dual-modality molecular imaging. Chem. Commun..

[98] Lee S.Y., Jeon S.I., Jung S., Chung I.J., Ahn C.H. (2014). Targeted multimodal imaging modalities. Adv. Drug Deliv. Rev..

[99] Cai W., Chen X. (2008). Multimodality molecular imaging of tumor angiogenesis. J. Nucl. Med..

